# Microstructured click hydrogels for cell contact guidance in 3D

**DOI:** 10.1016/j.mtbio.2023.100604

**Published:** 2023-03-10

**Authors:** Mariana I. Neves, Sílvia J. Bidarra, Mariana V. Magalhães, Ana L. Torres, Lorenzo Moroni, Cristina C. Barrias

**Affiliations:** a i3S – Instituto de Investigação e Inovação em Saúde, Universidade do Porto, Portugal; b INEB – Instituto de Engenharia Biomédica, Universidade do Porto, Portugal; c FEUP – Faculdade de Engenharia, Universidade do Porto, Portugal; d Department of Complex Tissue Regeneration, MERLN Institute for Technology-Inspired Regenerative Medicine, Maastricht University, Maastricht, Netherlands; e CNR NANOTEC - Institute of Nanotechnology, Università del Salento, Lecce, Italy; f ICBAS – Instituto de Ciências Biomédicas Abel Salazar, Universidade do Porto, Portugal

**Keywords:** Physical cues, Cell instructive, Protein sequestration, Cell therapy, Tissue engineering, Regenerative medicine

## Abstract

The topography of the extracellular matrix (ECM) is a major biophysical regulator of cell behavior. While this has inspired the design of cell-instructive biomaterials, the ability to present topographic cues to cells in a true 3D setting remains challenging, particularly in ECM-like hydrogels made from a single polymer. Herein, we report the design of microstructured alginate hydrogels for injectable cell delivery and show their ability to orchestrate morphogenesis via cellular contact guidance in 3D. Alginate was grafted with hydrophobic cyclooctyne groups (ALG-K), yielding amphiphilic derivatives with self-associative potential and ionic crosslinking ability. This allowed the formation of microstructured ALG-K_H_ hydrogels, triggered by the spontaneous segregation between hydrophobic/hydrophilic regions of the polymer that generated 3D networks with stiffer microdomains within a softer lattice. The azide-reactivity of cyclooctynes also allowed ALG-K functionalization with bioactive peptides via cytocompatible strain-promoted azide-alkyne cycloaddition (SPAAC). Hydrogel-embedded mesenchymal stem cells (MSCs) were able to integrate spatial information and to mechano-sense the 3D topography, which regulated cell shape and stress fiber organization. MSCs clusters initially formed on microstructured regions could then act as seeds for neo-tissue formation, inducing cells to produce their own ECM and self-organize into multicellular structures throughout the hydrogel. By combining 3D topography, click functionalization, and injectability, using a single polymer, ALG-K hydrogels provide a unique cell delivery platform for tissue regeneration.

## Graphical abstract

**Figure:**
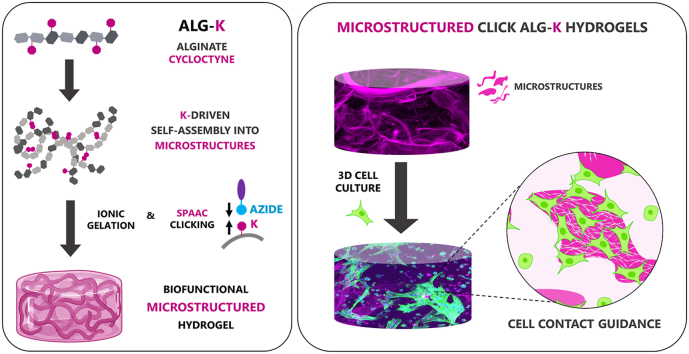

## 1 Introduction

The design of advanced biomaterials that mimic the extracellular matrix (ECM), a central component of the cell microenvironment in native tissues, remains a major goal of tissue engineering. Besides providing structural support, the ECM plays an active role in instructing cellular responses through well-orchestrated and precisely regulated biochemical and biomechanical signaling. In addition, it presents spatial heterogeneities that are determinant for tissue development, providing random or oriented topographic cues that inform cellular organization and dictate tissue architecture [[1], [2], [3], [4], [5]]. Nano- and micro-fabrication approaches have provided a variety of artificial substrates with geometrically controlled nano/microstructural features, which are valuable tools for studying the effect of physical cues on cellular contact guidance [5]. However, in such platforms topographical features are typically presented to cells in non-physiological 2D settings. The recreation of topographical features in 3D settings is an emergent field and their role on cell behavior has been largely overlooked. Strategies to address this need include the use of sandwich cultures [6,7], which provide quasi-3D environments, bridging the gap between 2D and 3D conditions. Other approaches explored the use of micrometric constructs as cell carriers for topography- and/or geometry-directed bottom-up tissue assembly [8]. To impart some anisotropy to conventional injectable hydrogels, which allow for minimally invasive cell delivery, these may be combined with polymeric microelements that act as discreet cell guidance cues [9,10]. For example, the inclusion of short magnetic-responsive microelements in soft hydrogels generated sophisticated injectable biomaterials with the ability to direct neuronal cell growth and orientation [9,10]. Remarkably, even if presented to cells in a random fashion, the inclusion of stiffer microelements inside an hydrogel matrix provides a way to guide cellular organization via mechanosensing mechanisms [11]. Still, most strategies reported so far rely on the use of hybrid systems combining different components, which brings complexity to the approach and may hamper clinical translation [12].

To address this limitation, we propose a radically different approach to create microstructured injectable hydrogels made from a single polymer, which should provide a much simpler setting for therapeutic cell delivery. We used alginate (ALG) as the hydrogel-forming polymer, which is amongst the most widely used biomaterials for designing artificial ECMs and vehicles for cell therapy [13,14]. Apart from capturing key features of the native ECM in terms of 3D structure, permeability and compliance, ALG hydrogels are also well-defined, bioinert, and amenable to chemical/physical modification. Thus, unlike ECM-derived hydrogels, they provide ideal “blank slate” materials for matrix engineering, allowing for precise customization of biochemical/mechanical properties and fine-tuning of cell-responsive/instructive features [13]. Herein, the hydrophilic ALG backbone was chemically modified with hydrophobic cyclooctyne groups, generating amphiphilic derivatives (ALG-K) with self-associative potential. Hydrophobic interactions between cyclooctyne groups drive the establishment of intra- and inter-chain associations, which, for high modification degrees (MD), assemble into stable microstructural domains. ALG-K derivatives also retain the ability to undergo ionic crosslinking, forming smooth or microstructured hydrogels at low MD (ALG-K_L_) and high MD (ALG-K_H_), respectively. These can be designed as injectable formulations, either as *in situ*-crosslinkable hydrogels or as micro-gels, as shown herein, allowing minimally invasive cell delivery. Cyclooctyne groups were strategically selected as the hydrophobic moieties as they are also highly reactive to azide-functionalized compounds, providing a versatile approach for hydrogel functionalization via strain-promoted azide-alkyne cycloaddition (SPAAC) [15,16]. Because SPAAC reactions are fast, bio-orthogonal, and cytocompatible, they can be carried out in the presence of cells, allowing hydrogels to be functionalized, namely with bioactive peptides, at any time during culture [17]. Interestingly, the hydrophobic character of ALG-K hydrogels also confers them the ability to retain proteins inside the network.

We show that mesenchymal stem cells (MSCs) embedded in microstructured ALG-K hydrogels can mechano-sense the stiffer microstructural domains, which act as physical anchors to promote cell attachment and to drive local changes in cell shape from round to elongated. This allows MSCs to exert tension, secrete their own ECM, and finally assemble into extensive multicellular networks, guided by the 3D topography of the hydrogel matrix.

## 2 Materials and methods

### 2.1 Production of alginate-cyclooctyne derivative (ALG-K)

Ultrapure ALG (mannuronate-to-guluronate content 51%, Pronova UP LVM, FMC BioPolymers) carboxyl groups were modified with N-(1*R*,8*S*,9*s*)-Bicyclo6.1.0non-4-yn-9-ylmethyloxycarbonyl-1,8-diamino-3,6-dioxaoctane (BCN-amine, Sigma-Aldrich) by carbodiimide chemistry. Briefly, ALG was dissolved (1 w/v%) in MES buffer saline (0.1 ​M MES, 0.3 ​M NaCl, pH 6.5). N-Hydroxysulfosuccinimide sodium salt (sulfo-NHS, Sigma-Aldrich) and (3-Dimethylaminopropyl)-N′-ethylcarbodiimide hydrochloride (EDC, Sigma-Aldrich) were sequentially added (1:2 ​M ratio) to the ALG solution. EDC:COOH molar ratios of 1:20 (5% activation) and 1:10 (10% activation) were used to produce ALG-K derivatives with low (ALG-K_L_) and high (ALG-K_H_), respectively. BCN-amine dissolved in dimethyl sulfoxide (DMSO) and deionized water (dH_2_O) (5:2 v/v) was added in a 1.2 ​M excess to EDC. The reaction was performed under inert atmosphere (Ar) and room temperature (RT) for 20 ​h. To stop the reaction, hydroxylamine (Sigma-Aldrich) was added in equimolar ratio to EDC. Polymer solutions were dialyzed for 3 days in dH_2_O with decreasing concentrations of NaCl (0.75–0 ​wt%), freeze dried and stored (−20 ​°C). Control materials were produced following this protocol without BCN-amine addition.

### 2.2 Analysis by nuclear magnetic resonance (1H NMR)

The successful incorporation of BCN-amine onto ALG was determined by 1H NMR. Samples were dissolved at 0.8 ​wt% in deuterium oxide (D_2_O, Sigma-Aldrich) and 3-(trimethylsilyl)propionic-2,2,3,3-d4 acid sodium salt (TSP-d4, Euriso-top) was added as internal standard. Spectra were recorded on a BRUKER AVANCE III (400 ​MHz, 9.4 ​T) NMR spectrometer and analyzed using Mnova software (version 11.0, Mestrelab Research). MD was determined correlating the area of ALG peaks (*δ* ​= ​3.5–4.3 ​ppm) with the new BCN-amine peak (*δ* ​= ​2.18–2.35 ​ppm), according to the following equation (1):

(1) $$MD=\left(\frac{\frac{\int \delta BCN}{H_{\delta BCN}}}{\frac{\int \delta ALG}{H_{\delta ALG}}+\frac{\int \delta BCN}{H_{\delta BCN}}}\right)x\;100\;\left(\%\right)$$

where integrals correspond to the area of the peaks in a particular *δ* and H corresponds to the number of protons attributed to those same peaks.

### 2.3 Hydrophobic properties of ALG-K derivatives

For optical contact angle (OCA) measurements, films of 2 ​wt% polymer in dH_2_O were prepared by spin-coating in poly-d-lysine (PDL)-coated coverslips and left drying overnight (ON). A 4 ​μL drop of water was dispensed directly on top of dried films and OCA was measured on the first timeframe of contact with the surface, using the tangential method. A safranin staining was performed after analysis to ensure the presence of a uniform alginate film. To assess the presence of hydrophobic domains, ALG-K solutions (0.5 ​wt%) were prepared in dH_2_O or 0.9% NaCl (saline) and plated into a fluorescence plate. Then, 8-Anilino-1-naphthalenesulfonic acid (ANS, 0.5 ​mM in dH_2_O) was added in a 50/50 proportion and fluorescence spectra (λ_ex/em_: 370/400–600 ​nm) were recorded. For determining critical aggregation concentration (CAC), ALG-K solutions were prepared in dH_2_O and sequentially diluted down to 0.001 ​wt%. Coomassie blue (Bluesfate, NZYTech) was added in a 50/50 proportion. CAC was confirmed by OD change in the absorbance of Coomassie blue (λ ​= ​618 ​nm) [18].

### 2.4 Viscometry

Manual viscosity (1 s^−1^ shear rate) and shear rate ramp (0.1 s^−1^ to 1000 s^−1^) tests of polymer solutions (2 ​wt% in dH_2_O) were performed using a Kinexus Pro Rheometer (Malvern), using a 40 ​mm diameter and 0.5° angle geometry at RT.

### 2.5 Production of 3D hydrogel discs by internal gelation

Hydrogel discs were prepared by internal gelation as previously described [19]. Briefly, a precursor solution was prepared at a final concentration of 1.5 w/v% polymer in 0.9 ​wt% NaCl. The solution was mixed with an aqueous suspension of sterile CaCO_3_ (CalEssence® 70 Enhanced Purity PCC) at a CaCO_3_/COOH molar ratio of 0.3 [19]. Then, a fresh solution of glucone delta-lactone (GDL) was added to trigger gelation. The CaCO_3_/GDL molar ratio was set at 0.25. Discs were prepared by dispensing 15 ​μL of the mixture into a Teflon-spacer-Teflon (500 ​μm height) sandwich system with a gelation time of 15 ​min. For electron microscopy imaging analysis, hydrogels were produced with 100% content of ALG-K. For all other studies, unless otherwise stated, hydrogels were produced using blends of ALG-K with unmodified ALG at 65/35 w/w% ALG-K_L_/ALG and 50/50 w/w% ALG-K_H_/ALG, as explained below.

### 2.6 Cryo-scanning electron microscopy (CryoSEM)

Hydrogels were produced and rinsed in dH_2_O shortly prior to analysis. Samples were rapidly cooled (slush nitrogen), fractured and sublimated (‘etched’) for 120 ​s at −90 ​°C. A coating of Au/Pd was sputtered for 60 ​s and samples were studied at −150 ​°C. Images were acquired using Scanning Electron Microscope with X-Ray Microanalysis and CryoSEM (JEOL JSM 6301F/Oxford INCA Energy 350/Gatan Alto 2500).

### 2.7 Microindentation

The effective Young's Modulus (Eeff), and the elastic (E′) and viscous (E″) components of the dynamic modulus of hydrogels was determined by microindentation (PIUMA, Optics11 Life). Probe calibration was performed in Tris-buffered saline supplemented with 7.5 ​mM CaCl_2_ (TBS-Ca, pH 7.4) and against a rigid glass substrate. Measurements were performed with both the sample and the probe immersed in the buffer at RT, using a spherical probe (radius ​= ​25 ​μm) and cantilever spring constant of 0.5 ​N/m. A matrix scan (15 ​× ​15 xy-arrays, 20 ​μm-distance between points) of the surface was performed using the dynamic mechanical analysis (DMA) mode (2 ​μm-depth indentations at 1 ​Hz). Data were fit to Hertz contact model using Piuma Dataviewer software (DataViewer V2.1.10, Optics11).

### 2.8 Protein retention studies

In these studies, albumin fluorescein isothiocyanate conjugate (Alb-FITC, Sigma-Aldrich, bovine) or fibronectin (FN, Sigma-Aldrich, human) previously tagged with Alexa-Fluor™ 647 NHS ester, tris(triethylammonium salt (Invitrogen, 1 ​h at RT) were used. Discs were prepared as described in section 2.5 but Alb-FITC (∼18 ​ng/μg polymer) or FN-647 (0.2 ​ng/μg polymer) was added to the gel-precursor solution. Discs were then incubated in 100x volume of TBS-Ca. For each time point, fluorescence readings were obtained directly in discs by area scan measurements (λ_ex/em_ ​= ​495/525 ​nm for Alb-FITC and λ_ex/em_ ​= ​651/672 ​nm for FN-647). Fluorescence images were obtained by ZOE Fluorescent Cell Imager (BioRad) using the same acquisition settings for all conditions.

### 2.9 SPAAC in gel-precursor solution (in-sol)

Polymer solutions (0.25–2.0 ​wt%) were prepared in 0.9% NaCl, plated and mixed (90/10 v/v%) with 3-azido-7-hydroxycoumarin (Coum-N_3_, ChemiMart GmbH) in DMSO, which only becomes fluorescent following SPAAC reaction (λ_ex/em_: 404/477 ​nm). Controls were mixed with the same volume of pure DMSO. Fluorescence was measured every 5 ​min for up to 8 ​h. Tested formulations were pure solutions or blends of 50/50 w/w% ALG-K_H_/ALG and 65/35 w/w% ALG-K_L_/ALG.

### 2.10 SPAAC in hydrogels (in-gel)

SPAAC in-gel was assayed in hydrogels produced either by internal gelation (section 2.5) or by external gelation (microgels). ALG and 50/50 w/w% ALG-K_H_/ALG microgels (2 ​wt% in 0.9% NaCl) were produced by extrusion under coaxial airflow using a Var J1 encapsulation unit (Nisco) into an isotonic 0.1 ​M CaCl_2_ solution for crosslinking, as previously reported [20]. After 10 ​min, microgels were rinsed in TBS-Ca. Hydrogels were incubated with sulfo-Cyanine3 azide (Cy3-N_3_, λ_ex/em_: 548/563 ​nm, ChemiMart GmbH) and/or Coum-N_3_ in Dulbecco's Modified Eagle Medium (DMEM) supplemented with HEPES buffer (pH 7.4) to mimic *in vitro* culture conditions. After rinsing unreacted azido-tag, hydrogels were incubated at 37 ​°C for at least 10 ​min, under static or dynamic conditions. To assess SPAAC cytocompatibility, cell viability (live/dead) and metabolic activity (resazurin) assays were performed after performing SPAAC in cell-laden microgels (8 ​× ​10^6^ MSC/mL). For live/dead microgels were incubated with calcein and ethidium homodimer for 30 ​min at 37 ​°C and then samples were imaged by confocal laser scanning microscopy (CLSM, Leica TCS SP5, Leica Microsystems). Cell viability was determined by the ratio of live cells to total number of cells (alive and dead) using Fiji software [21]. For metabolic activity, microgels were incubated with a 20 v/v% resazurin solution in culture medium and fluorescence was measured at λ_ex/em_: 530/590 ​nm after 2 ​h (37 ​°C). For both assessments, microgels that did not undergo SPAAC were used as controls.

### 2.11 Production of azide-functionalized peptide (RGD-N_3_)

RGD-containing peptide sequence (GGGGRGDSP, GeneScript) was modified with an azide group via the terminal amine group. Briefly, RGD was dissolved in sodium bicarbonate buffer (pH 8.3) and NHS–C3-azide in DMSO was added in equimolar amount. The mixture reacted for 4 ​h at RT with agitation. Azide incorporation was indirectly monitored by quantifying the consumption of free amines with the fluorescamine assay (Sigma-Aldrich). Fluorescence readings (λ_ex/em_: 400/460 ​nm) before and after reaction were compared with a glycine standard curve (Fig. S1), and the reaction efficiency was determined to be 81.07%.

### 2.12 Biofunctionalization of ALG-K with RGD-N_3_ via SPAAC

To access the ability to perform RGD-N_3_ coupling into ALG-K via SPAAC in-sol and in-gel and confirm the bioactivity of the grafted peptide, a cell adhesion study in 2D was performed with 3 types of hydrogel films: I) ALG-K (no peptide); II) ALG-K grafted with RGD-N_3_ (400 ​μM, ON, 20 ​°C with agitation) via SPAAC before film production (in-sol); and III) ALG-K grafted with RGD-N3 (800 ​μM, 3 ​h, 37 ​°C in static conditions) via SPAAC after film production (in-gel). Films were prepared by spin-coating ALG solutions on plastic coverslips, followed by immersion in 0.1 ​M CaCl_2_ for 10 ​min for crosslinking. After this, hydrogel films were stabilized in complete culture medium for 3 ​h. For in-gel SPAAC, the culture medium was supplemented with RGD-N_3_ and rinsed twice before seeding Human Wharton's jelly MSC (passage 6–10). MSCs were seeded on films at 2 ​× ​10^4^ ​cells/cm^2^ and cultured for 24 ​h in Minimum Essential Medium Eagle with no nucleosides (alfa-MEM), supplemented with 10% v/v fetal bovine serum (MSC qualified) and 1% v/v penicillin/streptomycin (all from Gibco). MSCs (labeled with calcein) attached to ALG-K (labeled with Coum-N_3_) were imaged by CLSM.

### 2.13 3D cell-laden hydrogels

For the 3D cultures, MSC-laden hydrogel discs were produced as described in section 2.5 with an additional step in which MSCs were added to the gel-precursor solution (1 ​× ​10^7^ ​cells/mL gel). Formulations containing grafted RGD-N_3_ underwent reaction in solution (800 ​μM, ON, 20 ​°C with agitation) prior to cell-embedding and hydrogel production. Gelation occurred for 30 ​min at 37 ​°C. In some experiments, the unmodified ALG fraction was replaced by ALG partially oxidized with sodium periodate (at 1% per monomer), as previously described [20]. Metabolic activity was evaluated using the resazurin assay (Merck). Discs were incubated with a 20 v/v% resazurin solution in culture medium and fluorescence was measured at λ_ex/em_: 530/590 ​nm after 2 ​h (37 ​°C). Data was normalized to the total number of cells at the endpoint, by dissolving discs with Trypsin-EDTA solution.

### 2.14 Cell morphology and ECM production by immunostaining

Hydrogels were fixed with 4% paraformaldehyde in HBSS (20 ​min, RT) and permeabilized with a 0.2% Triton X-100 in HBSS (20 ​min, RT). Samples were blocked in 1.5% bovine serum albumin (BSA) in HBSS (1 ​h, RT) and incubated with primary antibodies (1:100) rabbit anti-human fibronectin (FN, F3648, Sigma-Aldrich), mouse anti-human vinculin (VIN, V9131, Sigma), or mouse anti-human Yes-associated protein (YAP, sc-101199, Santa Cruz) and Phalloidin-488 (actin, 424201, Biolegend, 1:100) ON, at 4 ​°C. After washed, discs were incubated with secondary antibodies (donkey anti-mouse 488 A21202, goat anti-rabbit 488 A11008, goat anti-mouse 594 A11020, ThermoFisher Scientific, 1:500) and DAPI (nuclei, Merck, 3:500) for 1 ​h, washed and kept in HBSS. For ALG-K staining, SPAAC was performed before immunostaining, using fluorescent azide-tags (20 ​μM in culture medium) for 30 ​min (RT). Samples were immersed in Vectashield™ during image acquisition by CLSM. Image quantification was performed using Fiji software [21]. For cell spreading quantification, integrated density was measured using Z-stack max projections analysis after automatic Triangle thresholding. For the analysis of cell-cell interconnectivity degree, an image analysis workflow (in the format of a Fiji macro) was developed to quantify the degree of connectivity in the samples. The metric used reflects, for each image and on average, how many pixels are connected together. The analysis workflow starts with a maximum projection step to convert the image stack to a 2D image. Segmentation follows, using the standard Otsu's methods. All connected regions are then searched for, imposing a minimal size of 400 pixels (2.65 pixel per μm) to remove noise and/or artifacts. The average number of pixels in the identified connected regions is retrieved as the connectivity metric. Larger structures, even if in lesser numbers, lead to a higher connectivity metric. The results are normalized to the average values for the control samples.

### 2.15 Reverse transcription and real-time quantitative polymerase chain reaction

Hydrogels were collected at day 1, 7 and 14 of culture and dissolved in Trypsin-EDTA up to 10 ​min at 37 ​°C. Pellets were recovered by suspension and centrifugation in phosphate buffered saline (PBS, pH 7.4). RNA was extracted from hydrogels using the Quick-RNA MiniPrep (Zymo Research), following manufacturer's instructions. Then, RNA was reversed transcribed to single stranded cDNA using Takara cDNA synthesis kit. Quantitative Real-Time PCR (qRT-PCR) was carried out for at least 3 biological replicates for the target genes *CTGF*, *FN1*, *COL1A1*, and the reference gene *GAPDH* using as probe sets Hs.PT.58.14485164.g, Hs.PT.58.40986315, Hs. PT.58.15517795 and Hs.PT.51.1940505 (Applied Biosystems and Integrated DNA Technologies), respectively. Samples were run in duplicate using TaqMan master mix in an ABI Prism 7000 Sequence Detection System under the following conditions: 95 ​°C for 20 ​s, followed by 40 cycles at 95 ​°C for 3 ​s and 60 ​°C for 30 ​s. For the target genes *Runx2*, *ALP*, *OCN*, *COMP*, *ACAN*, qRT-PCR samples were run in triplicate using an iQ Sybr Green Supermix (Bio-Rad) in a CFX real-time PCR System (Bio-Rad) with the following conditions: 3 ​min at 95 ​°C, followed by 40 cycles at 94 ​°C for 30 ​s, 60 ​°C for 45 ​s and 72 ​°C for 30 ​s, and the last step at 55 ​°C for 10 ​s. The sequence of the primer pairs used are indicated in Supplemental Table S1. The expression value for each target gene was normalized to GAPDH value for all the samples.

### 2.16 MMPs activity in cell culture supernatants

Cell culture supernatants were analyzed for the presence of secreted MMPs by gelatine-zymography on day 14. Before sampling, cell-laden hydrogels were maintained overnight in serum-free medium. The conditioned media were collected and centrifuged (10000 ​rpm, 5 ​min) to remove cell debris, and loaded into gelatin-SDS polyacrylamide gels. Sample volumes were adjusted to yield equivalent total protein contents in the supernatant, which were quantified using the DC Protein assay (Bio-Rad). A protein ladder was run as a reference. The gel was run in 1x Tris–Glycine SDS running buffer at 80 ​V (Mini-Protean Tetra Cell system, Bio-Rad). After electrophoresis, gels were washed twice with 2% v/v Triton X-100 and incubated in MMP substrate buffer (50 ​mM Tris–HCl, pH 7.5, 10 ​mM CaCl2) for 16 ​h at 37 ​°C. Thereafter, gels were washed and stained with 0.1% w/v Coomassie Blue solution (Sigma). MMP proteolytic activity was visualized as clear bands against a blue background of stained gelatin substrate. MMP quantification was performed using Fiji software [21].

### 2.17 Statistical analysis

Statistical analysis was performed using GraphPad Prism software (GraphPad Software Inc., version 6.0). Data was analyzed by D'Agostino and Pearson omnibus normality test. Groups were compared using Welsh's *t*-test or Mann-Whitney test in case of parametric or non-parametric distributions, respectively. A confidence level of 99% was used and statistical differences are represented by ∗ (P ​< ​0.05), ∗∗ (P ​< ​0.01), ∗∗∗ (P ​< ​0.001) and ∗∗∗∗ (P ​< ​0.0001).

## 3 Results

### 3.1 Amphiphilic cyclooctyne-modified alginate derivatives

**Fig. 1:**
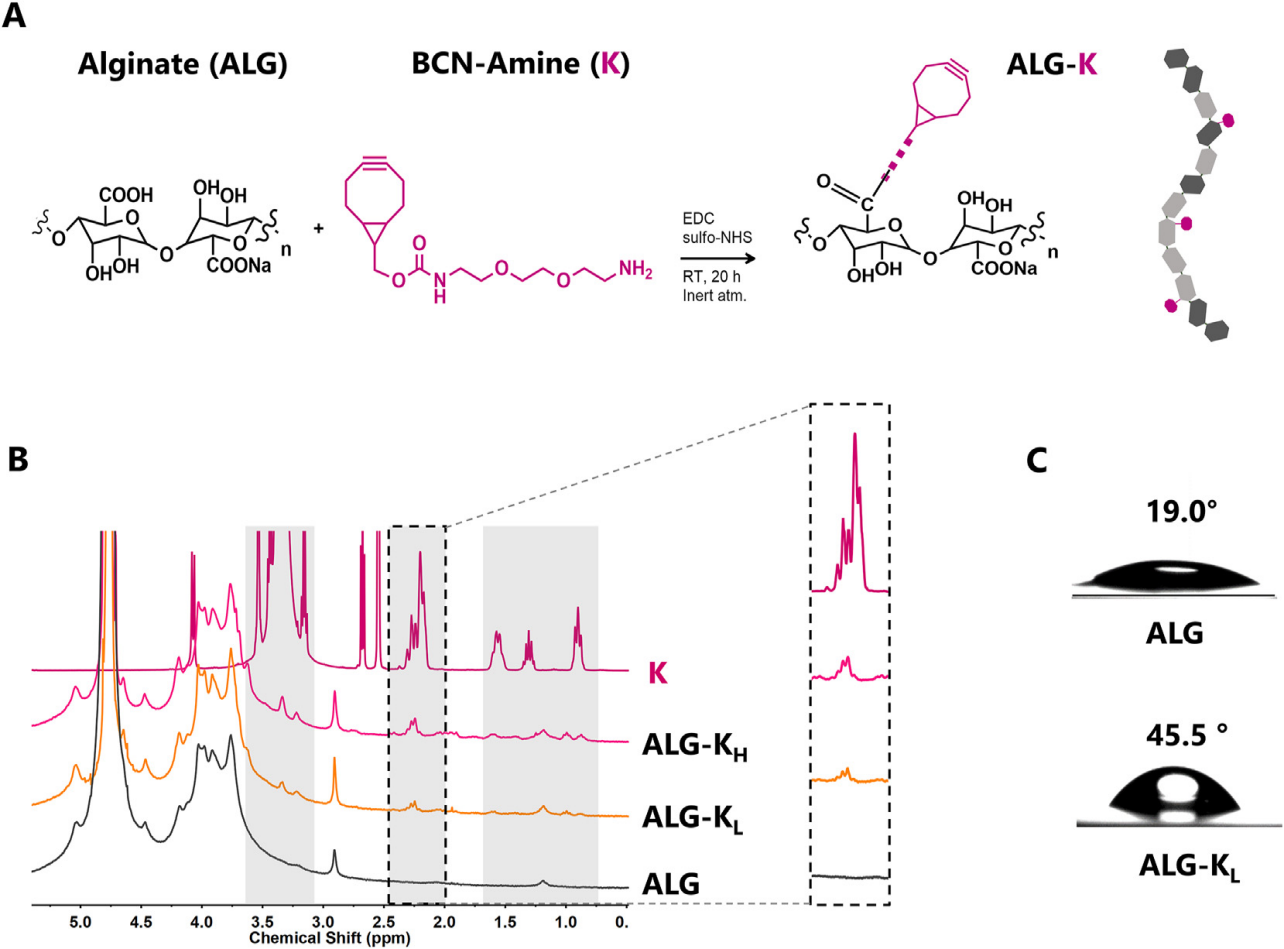
**Synthesis of amphiphilic cyclooctyne-modified alginate derivatives is efficient and reproducible. A)** Schematic representation of alginate-cyclooctyne derivative (ALG-K) production by BCN-amine (K) incorporation onto ALG carboxyl groups via carbodiimide chemistry. **B)** 1H NMR of ALG-K derivatives with low (ALG-K_L_; MD ​= ​2.7 ​± ​0.4%) and high (ALG-K_H_; MD ​= ​5.1 ​± ​0.8%) modification degrees (MD, average values from n ​≥ ​4 independent modification batches). Gray regions show characteristic K peaks, and the inset shows the one used for MDs calculation. **C)** Optical contact angles of ALG and ALG-K_L_ films.

**Fig. 2:**
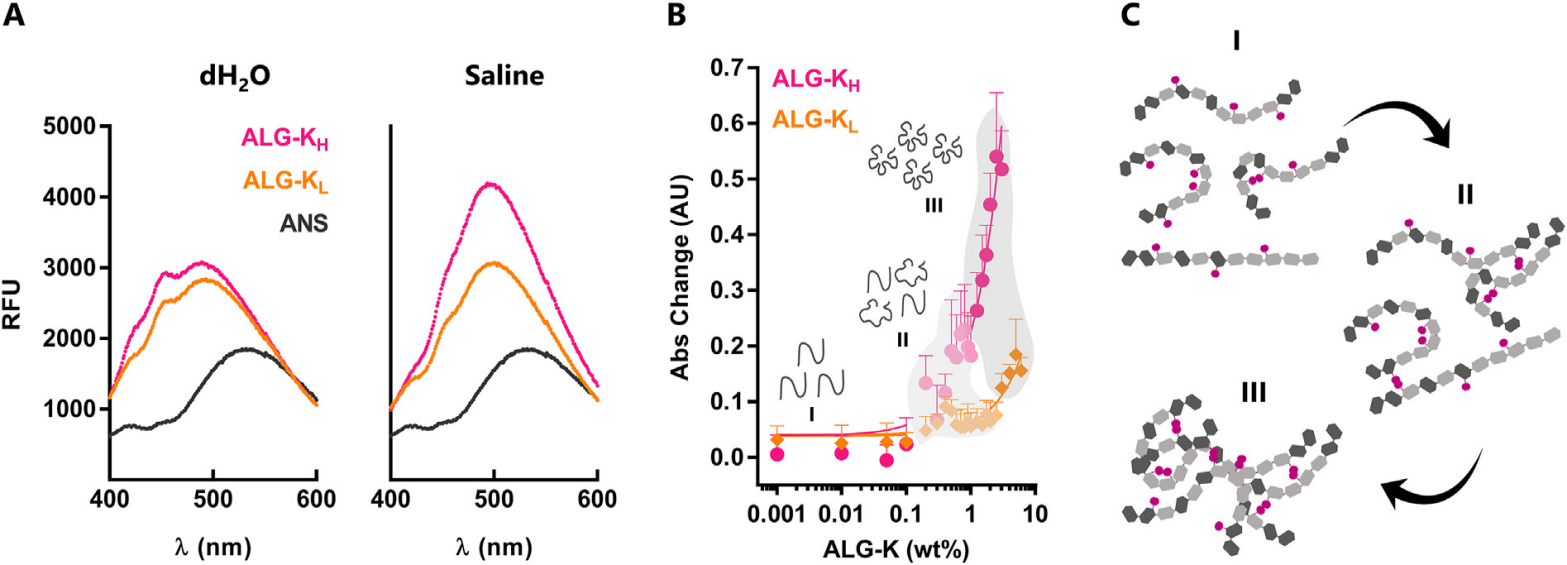
**ALG-K derivatives spontaneously self-associate in aqueous solvents. A)** Changes in ANS fluorescence spectra when mixed with ALG-K solutions in dH_2_O or 0.9 ​wt% NaCl (saline). **B)** Coomassie blue absorbance change (λ ​= ​618 ​nm) when mixed with ALG-K derivatives for CAC determination. **C)** Schematic representation of ALG-K hydrophobic inter- and/or intramolecular associations via cyclooctyne groups, which are improved by increasing MD and/or polymer concentration in solution. (For interpretation of the references to colour in this figure legend, the reader is referred to the Web version of this article.)

For the synthesis of SPAAC-clickable amphiphilic derivatives (ALG-K), alginate (ALG) was modified with different amounts of strained cyclooctyne groups (BCN-amine, K) by carbodiimide chemistry (Fig. 1A). Coupling was confirmed by 1H NMR, where ALG-K spectra showed new peaks, namely in the region of 0.9, 1.6, and 2.2 ​ppm, corresponding to BCN-amine (Fig. 1B, inset shows the peaks used for MD calculation). Depending on the degree of carboxyl group activation (5% or 10%), derivatives with MD of 2.7 ​± ​0.4% (ALG-K_L_) and 5.1 ​± ​0.8% (ALG-K_H_) (average ​± ​SD, n ​≥ ​4 independent batches) were obtained. As the MD increased, ALG-K became more hydrophobic. While ALG-K_L_ easily dissolved in water into clear and slightly viscous solutions, ALG-K_H_ typically formed more viscous gel-like fluids, in agreement with previous reports [22,23]. The less hydrophilic character of ALG-K derivatives was characterized by optical contact angle (OCA) measurements with water drops on polymer films (Fig. 1C). ALG had OCA of 19°, while ALG-K_L_ was less hydrophilic, with OCA around 45°. The gel-like ALG-K_H_ samples formed non-uniform films, precluding accurate OCA measurements (Fig. S2A). The increase on ALG-K hydrophobicity at higher MD was further demonstrated with the extrinsic hydrophobic probe ANS, which presents low fluorescence in aqueous solution (peaking around 540 ​nm) but is highly sensitive to the polarity of the nearest environment [24,25]. Thus, enhancement and/or spectral shifting of ANS fluorescence indicates interaction with hydrophobic moieties. As depicted in Fig. 2A, in presence of ALG-K derivatives in water, the ANS spectral peak shifted towards the blue region and the fluorescence intensity slightly increased. Such spectral effects were more pronounced for ALG-K derivatives in 0.9% NaCl, as the presence of cations partially shields the negative charge of carboxyl groups, decreasing repulsion between ALG-K molecules [26]. The higher proximity of polymer chains, and thus of BCN groups, likely favored the formation of hydrophobic domains that strongly interacted with ANS. In line with this, ALG-K_H_ solutions in saline were slightly cloudier than those prepared in water (Fig. S2B).

ALG-K derivatives at higher concentration and/or higher MD formed viscous to gel-like solutions in aqueous solvents. This suggests that hydrophobic-driven associations between BCN groups were occurring, as previously reported for other polymers [27]. To study the self-associative properties of ALG-K derivatives, we evaluated their critical aggregation concentration (CAC) through analysis of spectral changes of the Coomassie brilliant blue dye [18]. By plotting the absorbance (Abs) at 618 ​nm as a function of ALG-K concentration (Fig. 2B), we show that both derivatives lead to Abs changes, and that 3 main regions could be clearly distinguished: I) a low polymer concentration region, where changes in Abs were negligible; II) an intermediate concentration region, where Abs started increasing randomly; and III) a region where Abs increased abruptly and linearly with the polymer concentration, particularly for ALG-K_H_. The CAC values found for ALG-K_L_ and ALG-K_H_ were 1.73 ​wt% and 0.56 ​wt%, respectively, as estimated by the intersection of the two linear regression lines (Fig. S2C). Still, as shown in the plot, alterations of the molecular behavior ALG-K derivatives in solution start at much lower concentrations of ∼0.2 ​wt% for ALG-K_L_ and ∼0.1 ​wt% for ALG-K_H_. Collectively, these results support the occurrence of spontaneous intra/inter-molecular association of polymer chains via pendent hydrophobic 10.13039/501100011098BCN groups (Fig. 2C), as described for other hydrophobically modified ALG derivatives [18,28].

### 3.2 Rheological and mechanical properties of ALG-K derivatives

**Fig. 3:**
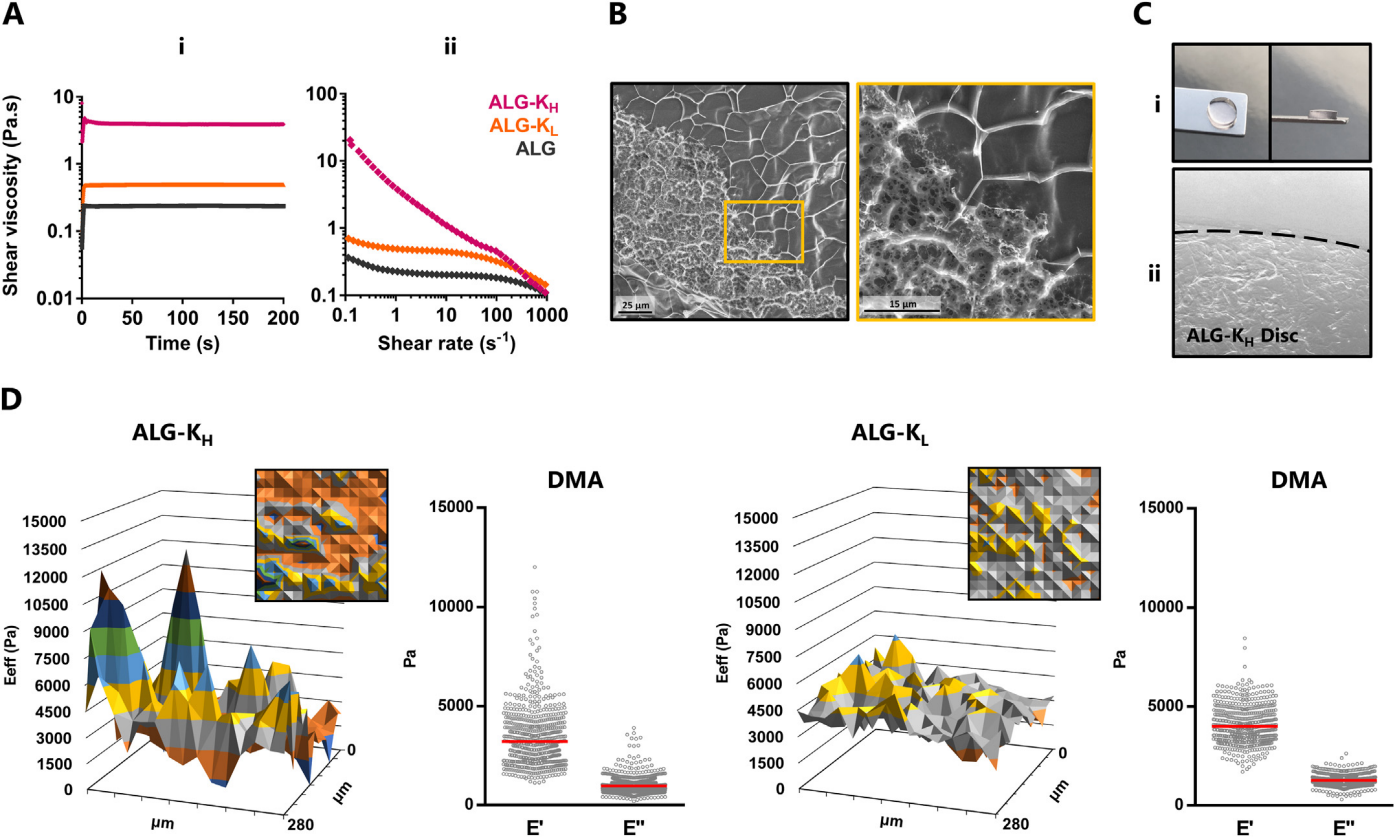
**ALG-K**_**H**_**hydrogels are microstructured and show a heterogeneous stiffness distribution. A)** Viscometry evaluation of ALG-K_H_ and ALG-K_L_ derivatives in comparison with ALG: (**Ai**) shear viscosity at 1 s^−1^ shear rate and (**Aii**) shear rate ramp. **B)** CryoSEM of ALG-K_H_ hydrogels, showing distinct denser/loser regions. The inset (magnification of yellow-squared region) allows to distinguish smaller pores within the denser mesh. **C)** ALG-K_H_ hydrogel discs (50/50 w/w ALG-K_H_/ALG): (**Ci**) the blend formulation allowed the production of transparent and stable ALG-K_H_ hydrogels by ionic internal gelation; (**Cii**) stereomicroscope image showing ALG-K_H_ hydrogels with microstructures. **D)** Surface mechanical properties obtained by static (effective Young Modulus, E_eff_) and DMA (elastic, E′, and viscous, E″, components) microindentation measurements. The DMA graphs show individual measurements (dots) and average values (bar) for E′ and E″ corresponding to 3 individual discs (produced independently, 225 measurements for each disc). (For interpretation of the references to colour in this figure legend, the reader is referred to the Web version of this article.)

We then evaluated the effect of hydrophobic-driven associations on the rheological and mechanical properties of ALG-K derivatives. Dynamic shear viscosity analysis showed that the presence of BCN groups increased the viscosity of ALG-K derivatives in comparison to unmodified ALG, particularly for ALG-K_H_ (Fig. 3Ai). As observed by shear rate ramp analysis, ALG-K_H_ solutions also presented gel-like profile with high shear-thinning behavior (Fig. 3Aii). Notably, ALG-K derivatives could still undergo calcium-initiated ionic gelation and ALG-K_H_ hydrogels showed a heterogeneous structure, with dense microstructural domains neighboring a looser network (Fig. 3B). This likely resulted from the spontaneous segregation of hydrophilic/hydrophobic regions, characteristic of hydrophobically-associating amphiphilic polymers, with consequent formation of hydrophobic microstructures [18]. In subsequent experiments, ALG-K derivatives were blended with unmodified ALG at adequate ratios (50:50 ALG-K_H_/ALG and 65:35 ALG-K_L_/ALG) to produce hydrogels with comparable SPAAC reactivity (explained in detail on section 3.4). We confirmed that microstructural domains were preserved in blended ALG-K_H_ hydrogels (Fig. 3C) and were absent in ALG-K_L_ hydrogels (data not shown).

The influence of the in-gel microstructures on the local mechanical environment of hydrogels was assessed by microindentation (Fig. 3D). ALG-K_H_ hydrogels showed spatial heterogeneity on effective Young Moduli (E_eff_) values, while ALG-K_L_ hydrogels were more homogeneous. This tendency was quantitatively confirmed by dynamic mechanical analysis (DMA, 1 ​Hz), which also showed the viscoelastic nature of the hydrogels. While ALG-K_L_ hydrogels presented an average E’ ∼4 ​kPa with lower dispersion, the stiffest regions of ALG-K_H_ hydrogels (E’ ∼12 ​kPa), which likely correspond to microstructures, were ca. 4-fold stiffer than the average value of the network (E’ ∼3.2 ​kPa). The two hydrogels showed a similar ratio of elastic (E’) to viscous (E”) moduli (Fig. S3).

### 3.3 Protein retention in ALG-K hydrogels

**Fig. 4:**
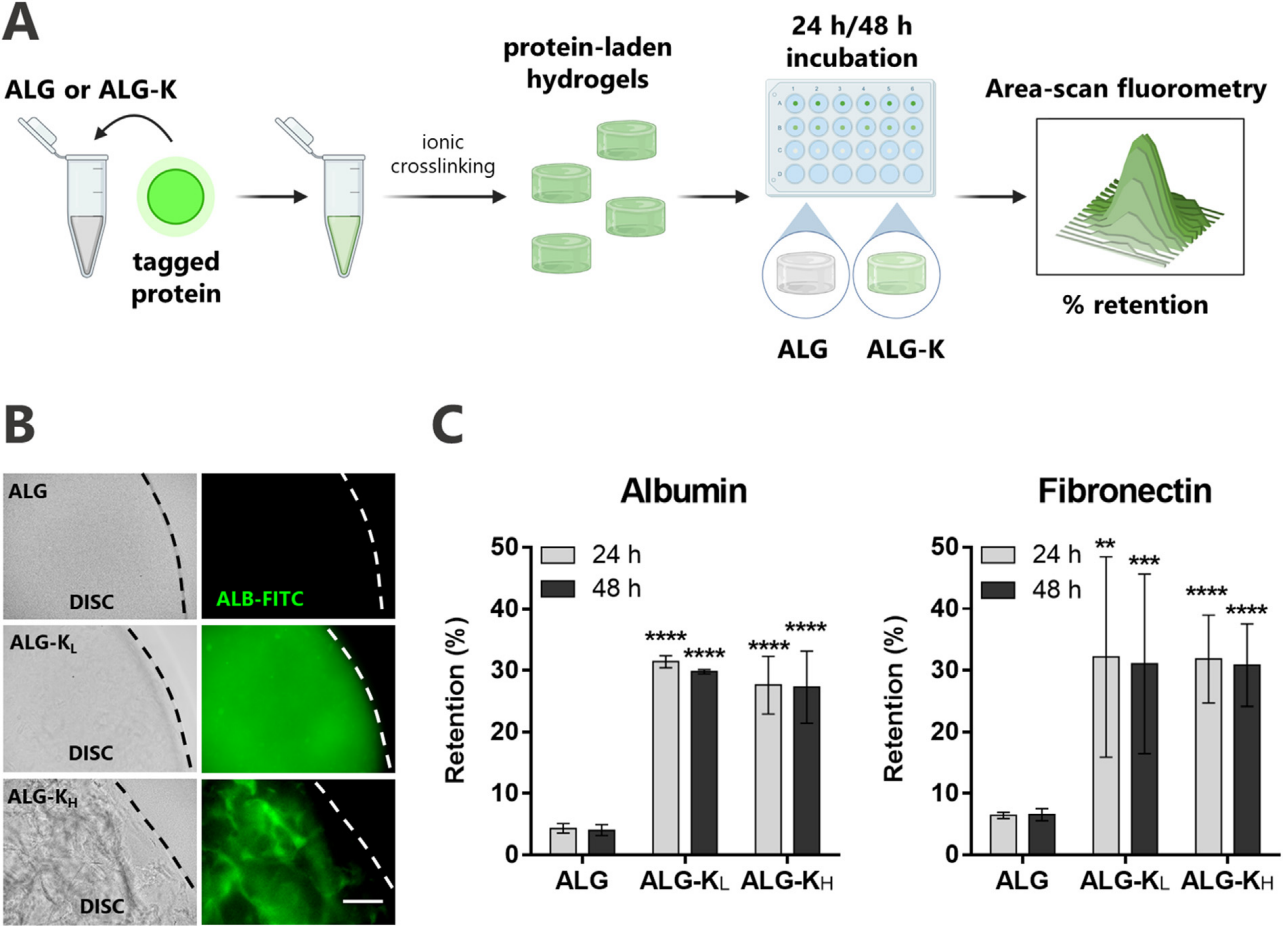
**ALG-K hydrogels improve protein retention. A)** Schematic representation of the experimental setup: polymer solutions were mixed with fluorescently labeled protein and hydrogels were produced by internal ionic gelation. Protein-laden hydrogels were incubated in a buffer solution and protein retention after 24 ​h and 48 ​h was directly measured in hydrogel discs by area scan fluorimetry. **B)** Brightfield and fluorescence images of hydrogel discs after 48 ​h of incubated buffer solution. **C)** Percentage of Albumin and Fibronectin retention in hydrogels after 24 ​h and 48 ​h, measured by spectrofluorimetry (n ​≥ ​3). Statistical differences are represented by ∗∗ (P ​< ​0.01), ∗∗∗ (P ​< ​0.001) and ∗∗∗∗ (P ​< ​0.0001) in comparison to ALG. No significant differences were found between ALG-K derivatives.

One key feature of hydrophobically-modified hydrogels is their ability to interact with hydrophobic compounds, or macromolecules with hydrophobic domains such as proteins, which are typically poorly retained inside hydrophilic networks. Protein-ALG-K interactions were probed using as model protein Alb-FITC that has hydrophobic domains and an isoelectric point around 4.8. Albumin is negatively charged at physiological pH, which induces electrostatic repulsion from polyanionic ALG chains fostering outward diffusion. Thus, it is typically released from ALG hydrogels within a few hours [22]. We hypothesized that this could be counterbalanced by hydrophobic interactions with ALG-K hydrogels, which in turn would delay release. The Alb-FITC was entrapped in disc-shaped hydrogels of unmodified ALG, ALG-K_H_/ALG (50:50), and ALG-K_L_/ALG (65:35) produced by internal ionic crosslinking. Protein retention along time was evaluated by fluorescence measurements (Fig. 4A). Fluorescence imaging of hydrogels after 48 ​h of incubation (Fig. 4B) showed that only ALG-K derivatives were able to retain visible amounts of Alb-FITC. Of note, while the overall intensity was similar and ALG-K_L_ discs were homogenously fluorescent, in ALG-K_H_ discs the fluorescent protein preferentially accumulated at the microstructures, which were clearly visible both in brightfield and fluorescence images. No apparent differences were detected on the swelling ability of ALG-K derivatives vs. unmodified ALG (data not shown). Spectrofluorimetric analysis of the whole discs (area scan mode) corroborated these results (Fig. 4C). While the unmodified ALG hydrogels become almost depleted of Alb-FITC after 24 ​h, both ALG-K derivatives retained ca. 30% of the originally loaded protein even after 48 ​h. Similar results were obtained for the retention of fibronectin (FN), a biologically relevant protein for cell adhesion, spreading and organization.

### 3.4 In-sol and in-gel SPAAC-functionalization of ALG-K

**Fig. 5:**
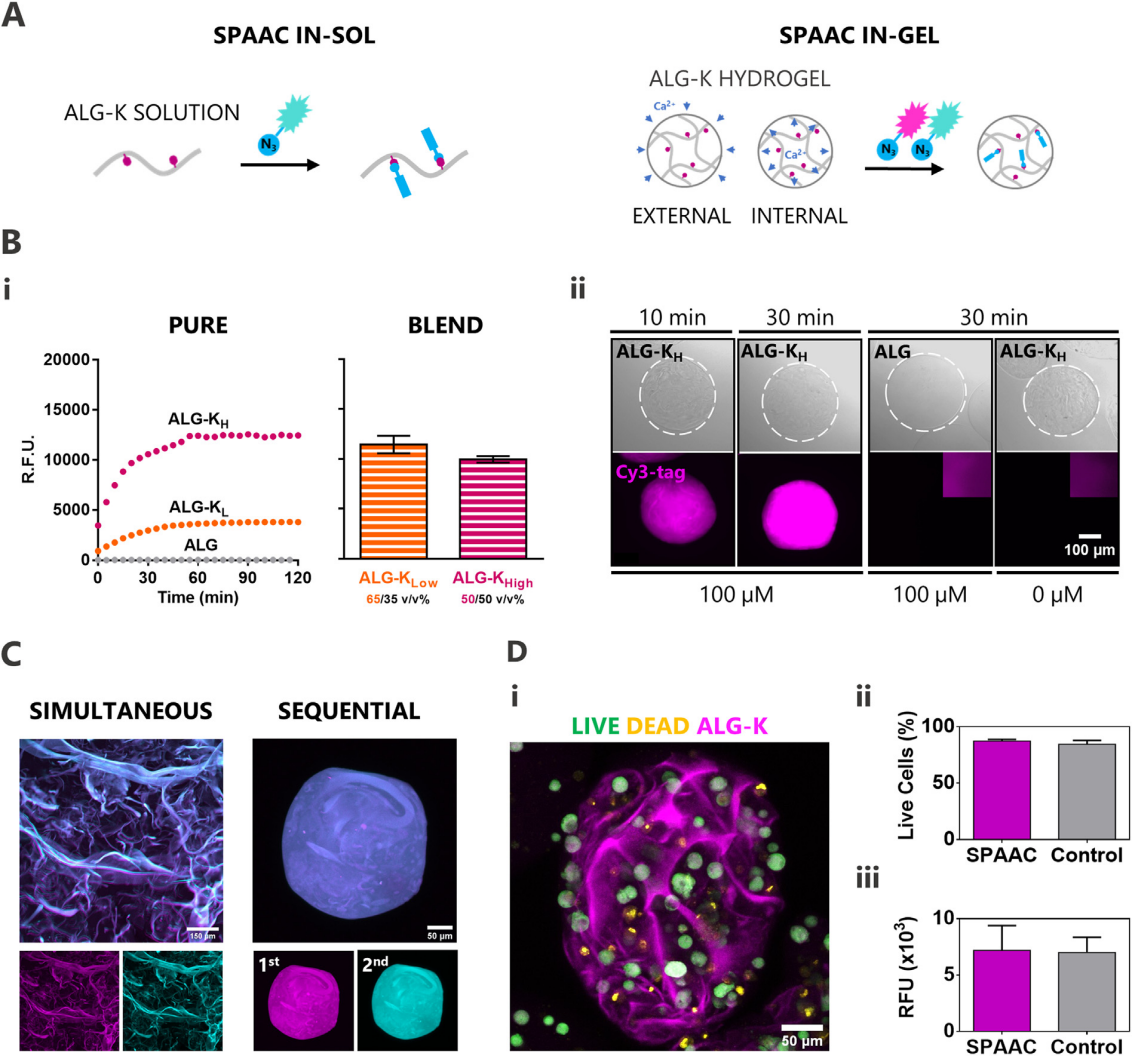
**SPAAC allows dynamic functionalization of ALG-K solutions and their hydrogels in the presence of cells. A)** Schematic representation of SPAAC strategy in solution (in-sol) and directly in hydrogels (in-gel). **B)** SPAAC in-sol and in-gel. (**Bi**) SPAAC kinetics in-sol (pure solutions and blends, at 0.5% and 1.5% wt.% polymer concentration, respectively) of ALG-K_L_ and ALG-K_H_ using a fluorescent azide-tag that only becomes fluorescent upon reaction. (**Bii**) SPAAC kinetics in-gel using a Cy3-fluorescent azido-tag in ALG-K_H_ microgels. **C)** Simultaneous and sequential SPAAC performed in-gel with two fluorescent-tags in hydrogels produced by either internal or external gelation, respectively, showing the ability to perform multiple functionalization steps. **D)** Cell viability (i and ii: Live/Dead assay – live cells in green, dead cells in yellow) and metabolic activity (iii, resazurin assay), before and after SPAAC reaction with Cy3 azido-tag (which rendered ALG-K fluorescent: in pink). (For interpretation of the references to colour in this figure legend, the reader is referred to the Web version of this article.)

After characterizing hydrophobic interactions of ALG-K derivatives, we further explored the azide-reactivity of strained cyclooctyne groups for biofunctionalization under mild conditions. SPAAC was performed both in gel-precursor solutions (in-sol) and in pre-formed hydrogels (in-gel) to illustrate the versatility of the strategy (Fig. 5A). In addition, ALG-K derivatives were tested in the form of microgels (external gelation) or *in situ*-forming hydrogels (internal gelation), as both can be used as injectable formulations. The click reaction was probed either using fluorogenic azido-coumarin (Coum-N_3_, which emits blue fluorescence only upon SPAAC conjugation) or a fluorescent azido-tag (Cy3-N_3_, red). For the in-sol reaction, Coum-N_3_ (in molar excess) was added to ALG and ALG-K solutions and fluorescence was monitored over time (Fig. 5Bi, pure). In both cases, successful SPAAC reaction was confirmed by the progressive increase in fluorescence intensity in presence of ALG-K derivatives, but not for unmodified ALG, with plateaus reached after ca. 1 h. The ALG-K_H_ solutions presented higher reactivity towards azides than ALG-K_L_, yielding higher fluorescence values. SPAAC reaction rates were also dependent on the polymer and azide-compound concentration (Fig. S4A). The difference between the two ALG-K derivatives was much less pronounced when they were tested at more concentrated gel-precursor solutions (1.5 ​wt% vs. 0.5 ​wt% alginate), likely because hydrophobic associations and microstructure-formation in ALG-K_H_ decreases the availability of cyclooctyne groups to react. Thus, we optimized the gel-precursor formulations, by blending ALG-K-derivatives with unmodified ALG, to yield comparable SPAAC-reactivity. This was verified for blends of 50:50 ALG-K_H_/ALG and 65:35 ALG-K_L_/ALG, as probed via reaction with Coum-N_3_ (Fig. 5Bi, blend).

**Fig. 6:**
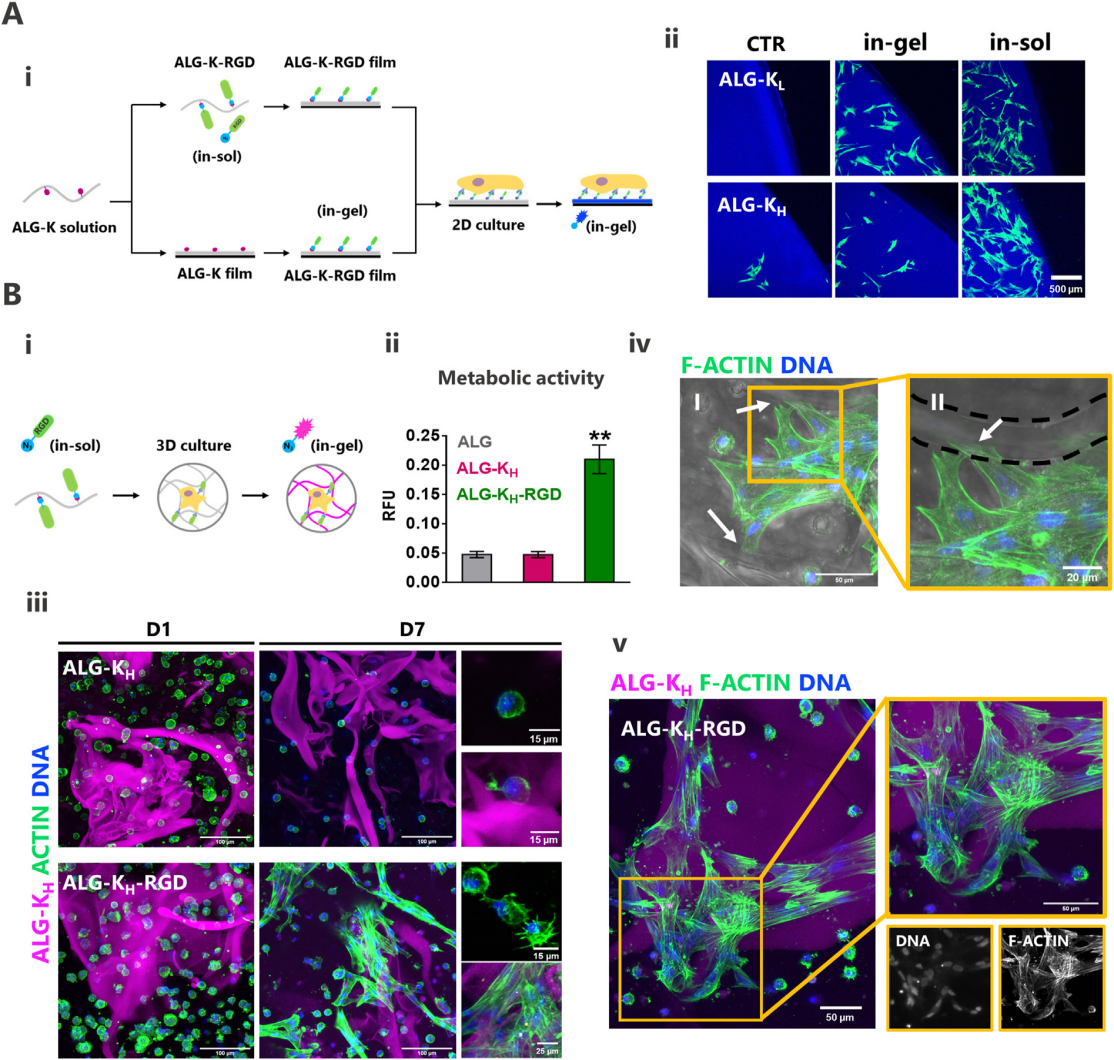
**MSCs attach and spread on microstructured regions of ALG-K**_**H**_**-RGD hydrogels. A)** 2D cell studies of ALG-K films. (**Ai**) ALG-K solutions were prepared and reacted with RGD-N_3_ before (in-sol) or after (in-gel) hydrogel film production. MSCs were seeded on films and cultured for 24 ​h. Before imaging, films were reacted with a blue-fluorescent tag (Coum-N_3_). (**Aii**) MSCs adhesion after 24 ​h in SPAAC-modified hydrogel films in comparison to non-modified (CTR). **B)** 3D cell studies of MSC-laden ALG-K_H_ hydrogels. (**Bi**) Gel-precursor solutions (50/50 ALG-K_H_/ALG) were reacted with RGD-N_3_ and MSCs were embedded within hydrogels produced by internal ionic gelation. At the end of culture, hydrogels were reacted with Cy3-N_3_ tags to stain the hydrophobic domains. (**Bii**) Metabolic activity (resazurin assay) of MSCs after 7 days of culture, normalized for the total number of cells. Statistical difference in relation to ALG and ALG-K_H_ is represented by ∗∗ (P ​< ​0.01). (**Biii**) MSCs morphology after 1 and 7 days in ALG-K_H_ and ALG-K_H_-RGD hydrogels. (**Biv**) Cell anchoring to ALG-K_H_ filaments at day 7. (**Bv**) Nuclei and actin staining showing cells alignment on Cy3-stained hydrophobic domain at day 7. (For interpretation of the references to colour in this figure legend, the reader is referred to the Web version of this article.)

The progression of in-gel SPAAC reaction was further evaluated by incubating ALG-K_H_ microgels with Cy3-N_3_ (100 ​μM) for 10–30 ​min (Fig. 5Bii). Microgels became readily fluorescent with intensity increasing over time, whereas controls (i.e., ALG with Cy3-N_3_ and ALG-K_H_ without Cy3-N_3_) remained non-fluorescent, as expected. The SPAAC reaction also proceeded at lower concentration of the azido-tag (10 ​μM), and the kinetics was similar under static vs. dynamic conditions, suggesting that the tag could freely permeate the pre-formed 3D network for in-gel reaction (Fig. S4B).

To probe the ability to perform dynamic click chemistry, i.e*.*, clicking azido-compounds at different time points, we added the two azido-tags simultaneously or sequentially to ALG-K_H_ hydrogels formed by internal or external gelation, respectively (Fig. 5C). The reactions were confirmed in both settings, as double-clicked hydrogels emitted fluorescence in the two wavelengths. Finally, to confirm SPAAC reactions in the presence of cells, MSC-laden ALG-K_H_ microgels were reacted with Coum-N_3_ in culture medium. As illustrated in Fig. 5D, cell viability (i and ii) and metabolic activity (iii) remained high and essentially unchanged after in-gel click reaction, confirming the cytocompatibility and bioorthogonality of SPAAC in our setup.

### 3.5 SPAAC-functionalization of ALG-K hydrogels with RGD peptides

Since ALG hydrogels are intrinsically non-adhesive [13,14,28,29], both ALG-K derivatives were modified (in-sol or in-gel) before cell culture with N_3_-functionalized RGD peptides to promote integrin-binding. Successful in-sol click reaction was shown by 1H NMR analysis (Fig. S5), where the ALG-K-RGD derivative presented new spectral peaks corresponding to RGD-N_3_. RGD incorporation was determined to be 2.37% for ALG-K_L_-RGD and 3.25% for ALG-K_H_-RGD. The obtained values are in accordance with the results obtained by fluorescence-based quantification with azido-tags (Fig. 5Bi). In fact, we observed comparable SPAAC reactivity for blends of 50/50 for ALG-K_H_/ALG (3.25% x 0.50 ​= ​1.6%) and 65/35 ALG-K_L_/ALG (2.37% x 0.65 ​= ​1.5%), which corroborated the 1H NMR quantification. Finally, the bioactivity of the clicked RGD-N_3_ peptide was demonstrated by cell adhesion studies (Fig. 6Ai). For 2D studies, cells were seeded on films casted directly from ALG-K-RGD solution (in-sol SPAAC) or on films casted from ALG-K solution and then reacted with soluble RGD-N_3_ (in-gel SPAAC). In both settings, the grafted RGD-N_3_ clearly supported cell adhesion to ALG-K films, as compared to their non-modified counterparts (ALG-K with no RGD) (Fig. 6Aii).

### 3.6 3D MSC culture in RGD-functionalized ALG-K hydrogels

For 3D culture, MSCs were embedded in disc-shaped ALG-K_H_ hydrogels. These were functionalized with RGD-N_3_ before adding the cells (in-sol) and clicked with a Cy3-N_3_ after cell culture (in-gel) for the fluorescent tagging of microstructures (Fig. 6Bi). After 1 week in culture, MSCs within ALG-K_H_-RGD hydrogels showed much higher metabolically active (Fig. 6Bii) than in control hydrogels (ALG, ALG-K_H_ without RGD), further confirming the bioactivity of the clicked RGD. In ALG-K_H_ hydrogels with no RGD, MSCs presented a compact spherical shape (Fig. 6Biii), as typically observed in physically confined hydrogel environments [19,29], occasionally extending small protrusions when in contact with microstructures (in pink). Significantly, in ALG-K_H_-RGD hydrogels the microstructural domains acted as docks for cell attachment and spreading, radically changing MSCs’ morphology from round to flattened (Fig. 6Biii). In contrast, cells in the neighboring smooth lattice remained essentially round, with short cytoplasmic protrusions, as typically seen in RGD-modified ALG hydrogels [19,29]. Fig. 6Biv and 6Bv show in greater detail the morphology of MSCs attached to the microstructured regions. In these regions, MSCs adopted an elongated morphology with well-defined stress fibers (f-actin) and stretched nuclei, typically observed in 2D contexts, aligned along the microtopographical features, and formed multicellular clusters. These cells also stained for vinculin, an actin-binding protein implicated in cell-matrix focal adhesions (Fig. S6).

### 3.7 3D cell contact guidance in microstructured ALG-K hydrogels

**Fig. 7:**
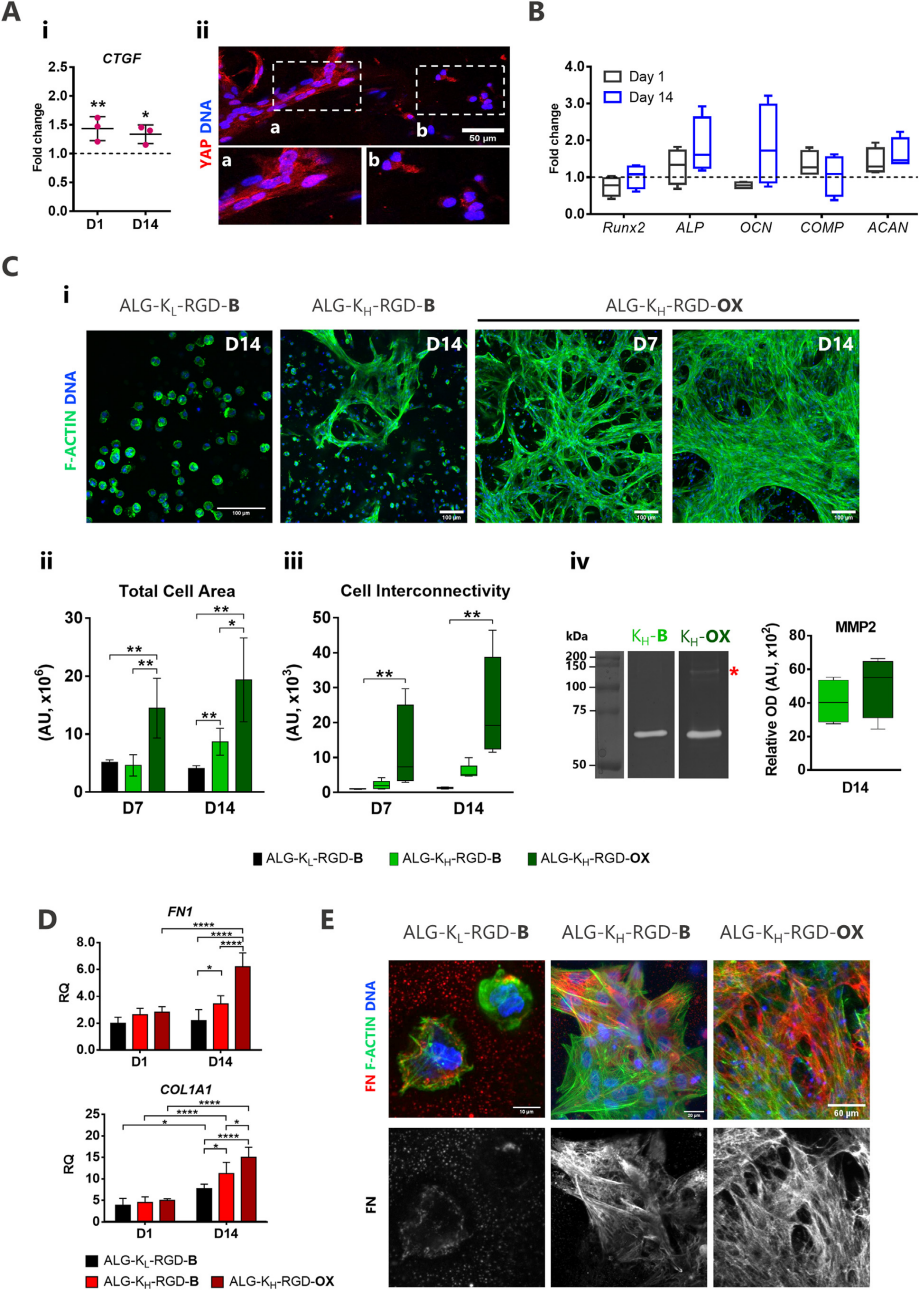
**MSCs assembled into multicellular clusters and produce more pericellular matrix within microstructured ALG-K**_**H**_**hydrogels. A)** Mechanosensing in microstructured hydrogels (**Aii**) Gene expression of CTGF represented as the fold change in expression levels of ALG-K_H_-RGD-B in comparison to ALG-K_L_-RGD-B. (**Aii**) Expression of YAP marker in MSCs embedded in ALG-K_H_-RGD hydrogels, showing higher translocation to the nuclei (in pink) in MSCs anchoring to the (**a**) microstructures when compared to MSCs (**b**) in the surrounding regions (day 7). **B)** Effect of the microstructures on the gene expression of osteogenesis and chondrogenesis markers, represented as the fold change in expression levels of ALG-K_H_-RGD-B in comparison to ALG-K_L_-RGD-B. **C)** MSCs spreading in non-degradable (B) versus degradable (OX) hydrogels. (**Ci**) Actin and nuclei staining showing differences in MSCs spreading ability in smooth hydrogels (ALG-K_L_-RGD-B), hydrogels with microstructural domains (ALG-K_H_-RGD-B) or combined with degradable (ALG-K_H_-RGD-OX) hydrogels. (**Cii**) Quantification of total cell area and degree of cell-cell interconnectivity (**Ciii**) obtained by image analysis. (**Civ**) MMP activity in conditioned media from MSC-laden ALG-K_H_-RGD-B and ALG-K_H_-RGD-OX hydrogels, assessed by gelatin-zymography. **D)** Gene expression analysis of ECM proteins (*FN1* and *COL1A1*) over time in culture. Bars represent mean values; error bars represent standard deviation (n ​≥ ​3). **E)** Differences in fibronectin (FN) production on day 14. Data shown for at least 3 individual experiments. Statistical differences are represented by ∗ (P ​< ​0.05) ∗∗ (P ​< ​0.01), ∗∗∗ (P ​< ​0.001) and ∗∗∗∗ (P ​< ​0.0001). (For interpretation of the references to colour in this figure legend, the reader is referred to the Web version of this article.)

To assess the effect of the 3D topographical cues on cell behavior, MSCs were cultured in smooth (ALG-K_L_-RGD) and microstructured hydrogels (ALG-K_H_-RGD). We started by evaluating the expression of the mechanosensitive *CTGF* (connective tissue growth factor) gene [30] that was significantly higher in ALG-K_H_-RGD compared to ALG-K_L_-RGD, (Fig. 7Ai). *CTGF* is a downstream target of the mechano-transductive transcriptional co-activator yes-associated protein (YAP) [31], and, thus, is consistent with the observation that elongated cells on the microstructural domains of ALG-K_H_-RGD hydrogels showed much higher YAP nuclear localization than round cells in the smoother neighboring lattice (Fig. 7Aii). Collectively, this data suggests that MSCs were effectively able to mechano-sense and respond to the stiffer microstructures inside the softer lattice. We also analyzed the effect of microstructures on the gene expression of osteogenesis (*RUNX2*, *ALP*, *OCN*) and chondrogenesis markers (*COMP*, *ACAN*), which are classical MSCs differentiation pathways. As shown in (Fig. 7B), MSCs in microstructured ALG-K_H_-RGD hydrogels showed slightly higher expression of *ALP* and *OCN* than ALG-K_L_-RGD at day 14, which may suggest some level of commitment to the osteoblastic lineage, although differences were not statistically significant.

For the analysis of cell morphogenesis, an additional test group was added, where the unmodified ALG (B, blank) fraction in ALG-K_H_-RGD hydrogels was replaced by degradable oxidized ALG (OX) to provide a more permissive lattice for neo-tissue formation. All the tested formulations, hereafter designated as ALG-K_L_-RGD-B, ALG-K_H_-RGD-B, and ALG-K_H_-RGD-OX, presented comparable amounts of grafted RGD (∼200 ​μM, previously optimized by our group [29,32,33] and others [28]). As depicted in Fig. 7Ci, the microstructural domains of ALG-K_H_-RGD-B hydrogels promoted MSCs clustering and spreading, which was not observed in ALG-K_L_-RGD-B hydrogels, even after 14 days of culture. In the more permissive ALG-K_H_-RGD-OX hydrogels, MSCs first clustered on the microstructural domains (day 7) and then assembled into interconnected multicellular structures throughout the hydrogel (day 14), guided by the 3D topography (Movie S1). Correspondingly, both the total cell spreading area (Fig. 7Cii) and the degree of cell-cell interconnections (Fig. 7Ciii) followed the trend: ALG-K_L_-RGD-B ​< ​ALG-K_H_-RGD-B ​< ​ALG-K_H_-RGD-OX. Over culture, cells in ALG-K_H_-RGD-OX hydrogels were able to partially remodel the hydrogel and contract the network, but without apparent disassembling of the microstructures. To better characterize this process, we analyzed the activity of matrix metalloproteinase-2 and -9 (MMP2 and MMP9, gelatinases) in secretomes from ALG-K_H-_RGD-OX vs. -B samples, which is intimately related to cell organization, tissue formation and remodeling. A band corresponding to MMP2 was detected in both samples (∼62–65 ​kDa) presenting overall higher activity (n ​= ​4) in the ALG-K_H-_RGD-OX. This condition additionally presented a high molecular weight band (∼130 ​kDa) which may represent a complex of MMPs (likely MMP9) with a tissue inhibitor of MMPs (TIMP, likely TIMP1) [34]. Overall, the profile of MMP expression is consistent with higher proteolytic activity in the ALG-K_H-_RGD-OX condition.

Supplementary data related to this article can be found at https://doi.org/10.1016/j.mtbio.2023.100604.

The following is the supplementary data related to this article:

Multimedia component 2 Multimedia component 2

Cell clustering, which was observed only in microstructured hydrogels, is known to stimulate cells to produce and organize ECM proteins into a fibrillar matrix, preceding neo-tissue formation [35]. To assess the effect of the microstructural elements on ECM production, we quantified the expression of *FN1* and *COL1A1,* which encode for two relevant interstitial matrix proteins – fibronectin and collagen type I, respectively (Fig. 7D). Overall, the expression of *FN1* and *COL1A1* increased from day 1 to day 14 in all conditions but was significantly higher in ALG-K_H_-B vs. ALG-K_L_-B and in ALG-K_H_-OX vs. ALG-K_H_-B groups. Corroborating these results, a much higher extent of FN pericellular deposition was detected in microstructured ALG-K_H_ (-B and -OX) hydrogels in regions with the clustered cells (Fig. 7E).

## 4 Discussion

In this study, we describe a new strategy to impart microstructural features to injectable hydrogels made from a single polymer, which provides a simple but powerful approach to guide morphogenesis by cell contact guidance in 3D. By grafting hydrophobic cyclooctyne moieties (K) to ALG, we created amphiphilic derivatives (ALG-K) with self-associative potential and SPAAC reactivity. As shown for other hydrophobically-modified polymers, physical interactions between hydrophobic groups drove inter- and intra-molecular associations between the polymer chains. Accordingly, ALG-K derivatives in aqueous solution exhibited the typical rheological properties of hydrophobically associating polymers, namely a remarkable increase in viscosity, near zero shear rate and shear-thinning behavior. The high viscosity at low shear rates likely arose from the combination of polymer chain entanglement with the presence of hydrophobic associations [15,23,36]. As such junctions are typically labile, further increasing the applied stress triggered shear-thinning [15,23,36]. Under certain conditions, associative polymers may form more stable junctions leading to physically cross-linked gel-like networks [22]. Here, using the Coomassie Blue assay, we proved that above a certain threshold of polymer concentration and/or MD, ALG-K derivatives formed aggregates in solution, while retaining the ability to form ionically crosslinked hydrogels. Significantly, the high MD derivative (ALG-K_H_) formed ionic hydrogels presenting an heterogenous network, where the segregation between hydrophilic and hydrophobic molecular regions led to the formation of stable microstructural domains within a smoother lattice. This imparted some 3D topography to ALG-K_H_ hydrogels, where the randomly distributed microstructures ranged from a few up to several hundred micrometers and were stiffer than the surrounding network. Hydrophobic-driven chain segregation has also been reported for alkyl-modified ALG polymers, which self-associate to form stable hydrogel networks [22]. However, these alkyl-ALG derivatives showed impaired formation of typical egg box junctions upon ionic crosslinking. Other hydrophobically modified polymers, such as dextran, were also shown to aggregate in aqueous solution [37], suggesting that our strategy could potentially be adopted to other biomaterials, increasing its significance. Another relevant feature of hydrophobically-modified polymers is their ability to interact with hydrophobic compounds, as shown here for ALG-K hydrogels using the extrinsic ANS probe. This also provides an effective approach for promoting protein sequestration inside the network. Since they typically present a mesh size in the order of 10^1^–10^2^ ​nm [29], the release of entrapped proteins from unmodified ALG hydrogels generally proceeds fast after a pronounced initial burst. In contrast, the retention of proteins (Alb and FN) in ALG-K hydrogels was significantly improved and most likely driven by hydrophobic interactions [22]. In future studies, this feature may be further explored to promote rational sequestration of specific bioactive/therapeutic compounds inside the hydrogel, prolonging their local action on embedded cells.

As discussed, cyclooctyne groups were strategically selected as the hydrophobic moieties for this work, as they also provide SPAAC-reactive sites for easy functionalization with azide-functionalized compounds, namely bioactive peptides. Interestingly, the reversible associations between pendent cyclooctyne groups in SPAAC-clickable PEG has been previously exploited as a strategy for tuning hydrogel mechanical properties [27], but not to produce microstructured networks as reported herein. We confirmed the SPAAC reactivity of ALG-K derivatives towards azide-bearing compounds, namely RGD peptides, not only in-sol as previously described [28] but also in-gel and in the presence of cells. In 3D MSC cultures, physical signals (stiffer microstructures) and biochemical cues (RGD) acted in concert to instruct cell behavior. The presence of RGD enhanced the metabolic activity of MSCs embedded in ALG-K_H_ hydrogels and potentiated the contact-guidance effect of the microstructural domains, as RGD ligands were required for effectively engaging the cell adhesion apparatus. A similar behavior has been observed in hybrid soft hydrogels incorporating stiffer microgels, where RGD-microgels promoted stronger interaction with fibroblasts, and enhanced guidance, as compared to unmodified ones [38]. Still, MSCs failed to adopt an elongated morphology inside smooth hydrogels (ALG-K_L_), even in the presence of RGD, demonstrating that microstructural cues were a central requirement for guiding cellular organization in 3D. In microstructured ALG-K_H_-RGD hydrogels, cells adhering to the microstructured domains were able to spread, presenting an organized cytoskeleton with well-defined actin stress fibers, and stretched nuclei, likely induced by the higher local stiffness as compared to the surrounding lattice [38]. These cells showed nuclear shuttling of YAP, a transcriptional regulator that changes subcellular localization in response to mechanical cues, supporting this hypothesis. Accordingly, MSC on microstructured hydrogels showed increased expression of *CTGF*, one of the most upregulated genes in response to mechanical stress applied to stromal cells [39]. Overall, this reflects the strong interaction of cells with microstructural domains and the underlying mechanical effect. Different studies have shown that substrate stiffness and topography jointly regulate stem cell specification, via different mechanisms, including YAP/TAZ signaling [40]. MSCs in microstructured ALG-K_H_-RGD hydrogels showed slightly higher expression of osteogenic markers*,* which may suggest some level of commitment to the osteoblastic lineage, but differences were not statistically significant. This was somehow expectable as cells were maintained in MSC-qualified serum, which inhibits differentiation. Also, these are bulk measurements that reflect the behavior of all the cells within the hydrogel and not just the smaller population of cells that binds to the microdomains. In future studies, single-cell RNA sequencing or spatial transcriptomics should provide helpful tools to better investigate these processes. Still, it should be highlighted that MSCs exhibit several therapeutic functions to support the repair and regeneration of injured tissues that do not require their differentiation into a specific lineage [41].

The final goal was to demonstrate that the presence of the 3D topographic cues could drive morphogenesis and neo-tissue formation by contact guidance. For this, ALG-K_H_ was blended with oxidized ALG to yield hydrogels with a more cell-permissive lattice [20,29]. In these hydrogels, the spatial heterogeneity provided by the microstructural domains could still be sensed by MSCs, guiding local adhesion, morphological alterations, and clustering. Remarkably, these clusters then acted as “seeds” for neo-tissue formation, orchestrating the organization of neighboring cells (located in the smoother regions) into multicellular networks. This empowered cells to produce their own ECM and to form extensive tissue-like structures throughout the hydrogels, illustrating their potential as injectable cell delivery vehicles for local tissue regeneration. Overall, these findings validate our approach to create 3D topography in hydrogels made from a single polymer, which afford cell contact guidance in 3D without the need to over-engineer the system, thus presenting clear advantages for clinical translation.

## 5 Conclusions

This work describes an innovative strategy for building microstructured hydrogels using amphiphilic alginate derivatives that self-associate via hydrophobic interactions and present SPAAC reactivity. Collectively, our results illustrate the versatility and relevance of our biomaterial platform, which provides i) 3D topography for cellular contact-guidance, driving MSCs’ morphogenesis into complex ECM-rich tissue-like structures; ii) cytocompatible click-based functionalization for modulating hydrogel properties in the presence of cells and over time; and iii) affinity for hydrophobic moieties for the local release/sequestration of bioactive compounds. This significantly leverages the properties of ALG hydrogels as artificial ECMs for cell delivery and tissue engineering. Also, the approach can potentially be applied to many other types of hydrogel-forming hydrophilic polymers, which expands its relevance.

## Author contributions

MIN: scientific conceptualization, experimental design, bench work, collected and analyzed data, manuscript writing. SJB: performed qPCR data collection and analysis. MVM: contributed rheology data collection and analysis. ALT: contributed to optimizing hydrogel formulations for 3D culture. LM: co-supervised the work and reviewed the manuscript. CCB: scientific conceptualization, designed and supervised the work, and reviewed the manuscript.

## Funding

This work was supported by project EndoSWITCH (PTDC/BTM-ORG/5154/2020) funded by 10.13039/501100001871FCT (Portuguese Foundation for Science and Technology). The authors thank 10.13039/501100001871FCT for CCB's IF research position (Grant No: IF/00296/2015), MIN's scholarship (Grant No: SFRH/BD/129855/2017 and COVID/BD/151886/2022), SJB’s research contract (DL 57/2016/CP1360/CT0006) and MVM’s scholarship (Grant No: SFRH/BD/10184/2022)

## Declaration of competing interest

The authors declare that they have no known competing financial interests or personal relationships that could have appeared to influence the work reported in this paper.

## Data Availability

Data will be made available on request.
