# Supplementary material for: Microstructured click hydrogels for cell contact guidance in 3D
Source: Mater Today Bio. 2023 Mar 10;19:100604. doi: 10.1016/j.mtbio.2023.100604 (PMC10034521; doi:10.1016/j.mtbio.2023.100604)
Supplement: Multimedia component 1 [file mmc1.pdf]

SUPPORTING INFORMATION

MICROSTRUCTURED CLICK HYDROGELS FOR CELL CONTACT GUIDANCE IN 3D

Mariana I. Neves[1,2,3], Sílvia J. Bidarra[1,2], Mariana V. Magalhães[1], Ana L. Torres[1,2], Lorenzo Moroni[4,5], Cristina C. Barrias[1,2,6,*]

[1] i3S – Instituto de Investigação e Inovação em Saúde, Universidade do Porto, Portugal

[2] INEB – Instituto de Engenharia Biomédica, Universidade do Porto, Portugal

[3] FEUP – Faculdade de Engenharia, Universidade do Porto, Portugal

[4] MERLN Institute for Technology-Inspired Regenerative Medicine, Maastricht University, Maastricht, the Netherlands

[5] CNR NANOTEC - Institute of Nanotechnology, Università del Salento, Lecce, Italy

[6] ICBAS – Instituto de Ciências Biomédicas Abel Salazar, Universidade do Porto, Portugal

***** corresponding author: ccbarrias@ineb.up.pt

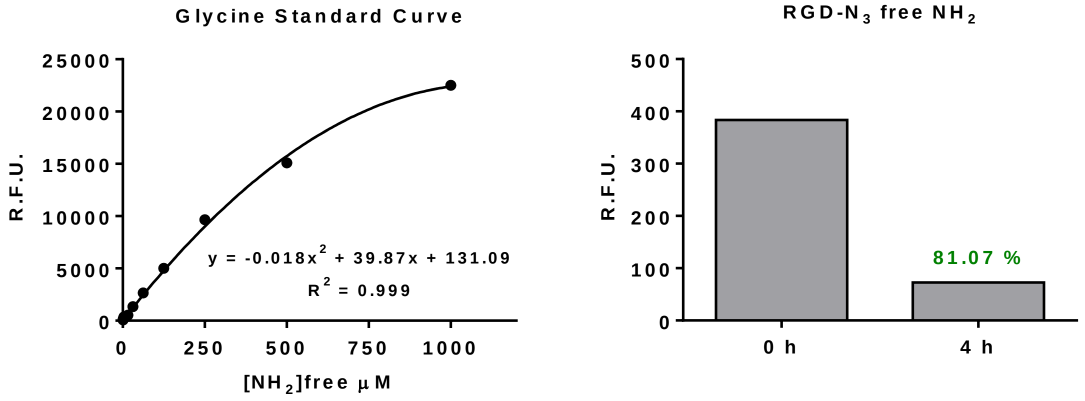

Figure S1. Glycine standard curve in fluorescamine assay and reduction of free-amines present in the RGD-containing peptide sequence after azidation reaction.

Table S1. Sequence of primer pairs for qRT-PCR

Gene symbol	Definition	Primer sequence (5'-3')
GAPDH	Glyceraldehyde-3-phosphate dehydrogenase	Fw: AGCCACATCGCTCAGACAC<br>Rev: GCCCAATACGACCAAATCC
ALP	Alkaline phosphatase	Fw: AGAACCCCAAAGGCTTCTTC<br>Rev: CTTGGCTTTTCCTTCATGGT
Runx2	Run related transcription factor 2	Fw: GTGCCTAGGCGCATTTCA<br>Rev: GCTCTTCTTACTGAGAGTGGAAGG
OCN	Bone gamma-carboxyglutamate protein or osteocalcin	AGAGTCCAGCAAAGGTGCA<br>TCAGCCAACTCGTCACAGTC
COMP	Cartilage oligomeric matrix protein	Fw: GCACCGACGTCAACGAGT<br>Rev: TGGTGTTGATACAGCGGACT
ACAN	Aggrecan	TACACTGGCGAGCACTGTAAC<br>CAGTGGCCCTGGTACTTGTT

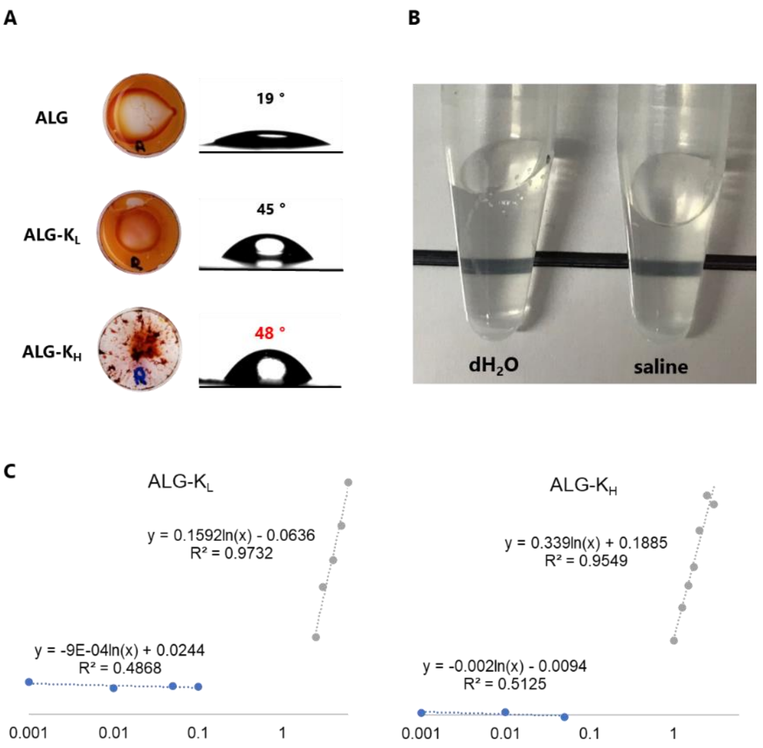

Figure S2. A) Safranin staining (orange) of ALG and ALG-K films produced for OCA analysis. ALG and ALG-K_L produced homogeneous films, evenly coating the coverslip, as opposed to ALG-K_H. This explains the low OCA differences observed between ALG-K_L and ALG-K_H, which retrieved an optical contact angle of 48 º. **B)** differences in translucency of ALG-K_H solutions prepared in dH2O or saline solution. **C)** linear transgressions used to determine CAC.

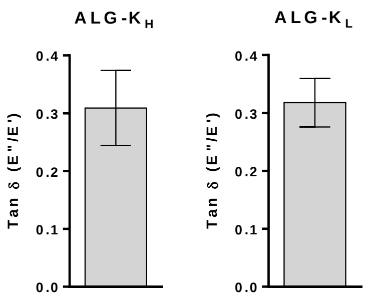

Figure S3. Tan δ (ratio E”/E’) of ALG-K_H and ALG-K_L hydrogels showing the proportion between elastic and viscous components is similar between both formulations.

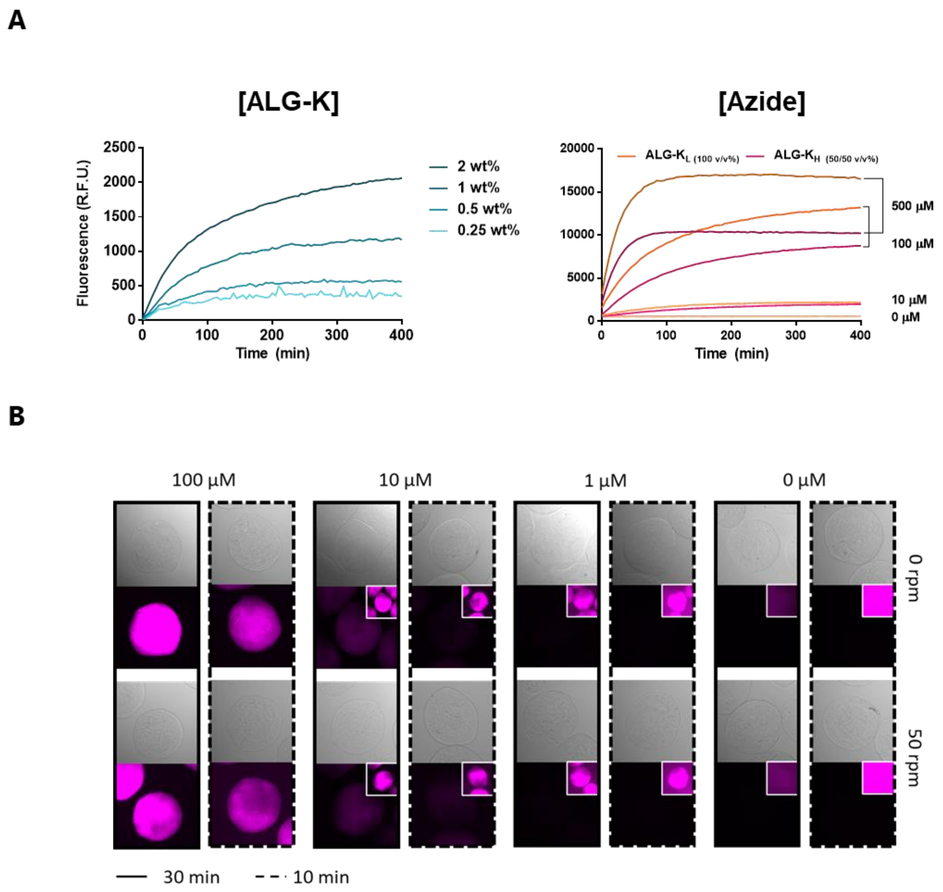

Figure S4. A) Effect of polymer concentration (0.25-2.00 wt% ALG-KH and 1 mM Coum-N_3) and azide-compound concentration (1.5 wt% ALG-KH, 0-500 µM Coum-N3) on the kinetics of SPAAC. **B)** Inverted microscopy images of ALG-K_H microspheres incubated with Cy3-N3, showing increase in fluorescence with time and Cy3-N3 concentration and no significant differences between static and dynamic conditions.

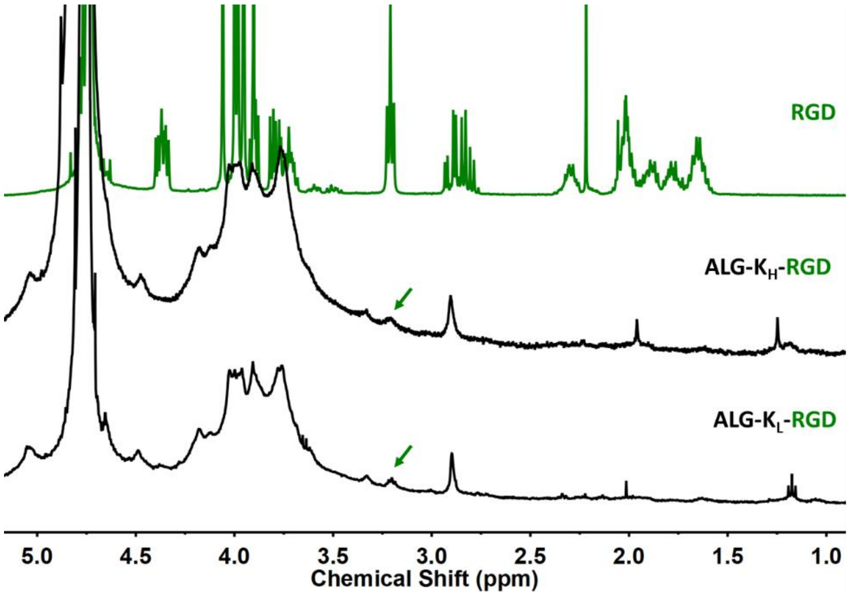

Figure S5. 1H NMR Spectra of ALG-K_H and ALG-K_L modified with RGD-N3 peptide (in-sol) reaction. Green arrows show RGD peak (δ = 3.14-3.25 ppm) used for RGD-incorporation quantification. ALG-K polymer solutions (2 wt% in 0.9% NaCl) were prepared and mixed with RGD-N3 (final concentration 400 µM) and allowed to react for 20 h at 20 ºC with agitation. After reaction, the solution was dialyzed against dH_2O using a D-TubeTM Dialyzer Maxi (MWCO 3.5 kDa, Milipore), frozen at -20 °C and freeze-dried.

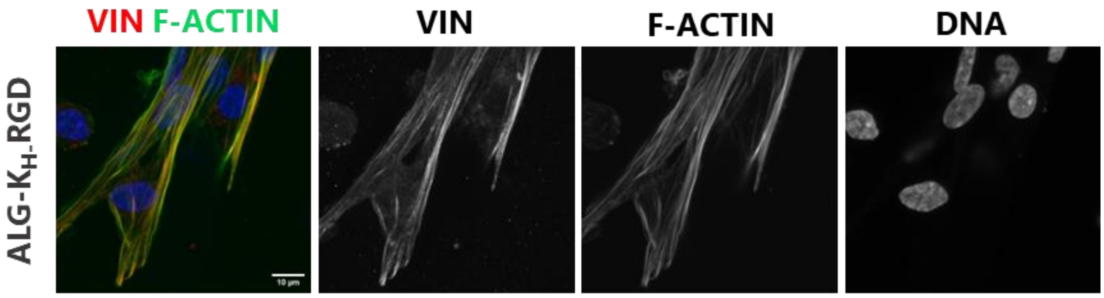

Figure S6. Immunostaining of MSCs embedded in ALG-K_H-RGD hydrogels showing vinculin accumulation in cell-microstructures contact regions at day 14.
